# Determinants of migraine headache among regular undergraduate students, of Wollo University, Dessie, Ethiopia: cross–sectional study

**DOI:** 10.1186/s12883-021-02466-4

**Published:** 2021-11-10

**Authors:** Mengesha Birkie, Mohammed Endris, Sintayehu Asnakew

**Affiliations:** 1 Department of Psychiatry, College of Medicine and Health Sciences, Wollo University, Dessie, Ethiopia; 2 Department of psychiatry, College of Medicine and Health Sciences, Wollo University, Dessie, Ethiopia; 3 Department of Psychiatry, School Of Medicine, College Of Health Science Debre Tabor University, Debre Tabor, Ethiopia

**Keywords:** Migraine headache, Wollo University, Students, Ethiopia

## Abstract

**Background:**

The prevalence of migraine headaches varied from 2.4 to 48.5% worldwide among university students. As per the knowledge of the investigators, the study of migraine headaches is not done in this study area. Hence, this study aimed to assess determinates of migraine headaches among regular undergraduate students, of Wollo University, Dessie, Ethiopia, 2020.

**Method:**

Institution-based cross-sectional study design was conducted from November 15–30 /2020 by using a self-administered questionnaire among 371 regular undergraduate students at Wollo University. A multistage sampling technique was employed to represent our study population. Identity migraine test, Migraine Disability Assessment Questionnaire, perceived stress scale, and Oslo social support scale was used. Descriptive statistics, binary and multiple logistic regression were employed. The adjusted odds ratio at a 95% confidence interval and level of significance (*p*-value < 0.05) was used to interpret the findings.

**Results:**

From 371 participants, the prevalence of migraine headache in this study was 34% (95% CI: 29.2, 38.5). Variables statistically associated with migraine headache, were a family history of headache (AOR = 3.83, CI: 2.313, 6.366), suicidal thoughts in the past 3 months (AOR = 10.76, CI: 2.117, 54.74), and had low perceived stress 62.6% (AOR = 0.374, CI: 0.205, 0.683).

**Conclusion:**

In this study, the prevalence of migraine headaches was very high. Family history of headache, suicidal thoughts, and low perceived stress were determinates for migraine headache. So special emphasis shall be given to those participants who had high perceived stress, a family history of migraine headache, and suicidal thoughts.

## Background

Migraine is an important health problem that affects more than 10% of the general population [1–3]. However, the prevalence varied from 2.4 to 48.5% worldwide among university students [4]. Although numerous studies had been conducted on the prevalence of migraine headaches within the general population, there were few studies among university students [5–9].

The prevalence of migraines among medical students ranges from 11 to 40% worldwide [10–12]. Moreover, migraine is the most common type of headache in young adults [13], and its frequency has been increased during student’s educational years of study [14]. The prevalence of migraine among university students was in Greece, 2.4% [15] and in Turkey, 12.4% [6] in Brazil, 25% [16], in china 7.91% [17], in Saudi Arabia 26.3% [18], in Iran 81.53% from all types of headache, of the 6.9% were suffering from migraine headache [19], in Jazan 5% [20], in Zahedan 7.14% [21] and Parakou University14.3% [22].

Several data on the epidemiology of migraines among young people and students are available in western countries [22–25]. Despite, in developing countries in Africa, especially in Ethiopia few data were available. The prevalence of migraine has been estimated to be 19.8% among Nigerian students [26], in Kenya 33.8% [27], and in Benin 11.3% [28]. Moreover, in studies conducted on students at the University of Gondar the prevalence of primary headache was 584 (81.11%), of them, 94(13.06%) were having a migraine [29].

Factors trigger migraine headaches such as stress, exposure to the sun, sleeplessness, eating habits, changes in weather conditions, temperature, frequent traveling, food items, oral contraceptives, and physical activities. Of them, the most common migraine triggers are lack of sleep and fatigue along with smoking [30–32]. Furthermore, having a family history of migraine, those students who enrolled in the second academic year, fatigue, anxiety, depression, stress, prolonged tension, changes in sleep or lack of sleep, hangovers, medications to treat other disorders, overuse of pain medications, panic attacks, strokes, dehydration, influenza, hypertension, strong odors, allergies, excessive intake of caffeine, caffeine withdrawal, smoked meats, skipping a meal, mechanical factors such as neck strain and cigarette smoking [18, 33].

Likewise the study conducted, in Iran University students, stress 63%,sunlight 55.6%,noise 48.1%,fatigue 77.8%,menstruation in women 55.6%,too much work and late sleep at night 40.7%,exercise(< 10%),smells like cigarette smoke and perfume odor 37%, and some foods like fatty and fried foods 33.3% [19]. Another study in Zahedan shows that the most common migraine triggers were Stress, 73% Sleep lack 52.5%, Reading 39.25%, and Fasting 39.55% [21] were the risk factors for migraine headaches.

Migraine headaches hurt individuals’ performance, and high costs are imposed on society due to its absenteeism in the workplace as well as education [34–36]. And also migraine headache has impacts on psychological problems such as depression, Obsessive-compulsive disorder, Bipolar and anxiety disorders [37]. Migraine headaches are under-diagnosed and treated conditions in undergraduate students. This leads to the compromised academic performance of students. Based on our knowledge, there are no studies done previously, about migraine headaches among Wollo university students. Therefore, this study aimed to assess the determinates of migraine headaches among regular undergraduate students in Wollo University, Dessie, Ethiopia.

## Methods and materials

### Study design, period, and setting

An institutional-based cross-sectional study was conducted among regular undergraduate Wollo university students from November 15–30, 2020. Wollo University is one of the thirteen 3rd generation universities which were established in 1997 E.C. It is located in the Amhara region, 401 KM away from the capital city of Ethiopia. The university was starting to work with the enrollment of 749 students in one faculty of education and currently, it has more than 7000 students with six colleges.

### Study participants, sample size determination, and sampling technique

**Fig. 1:**
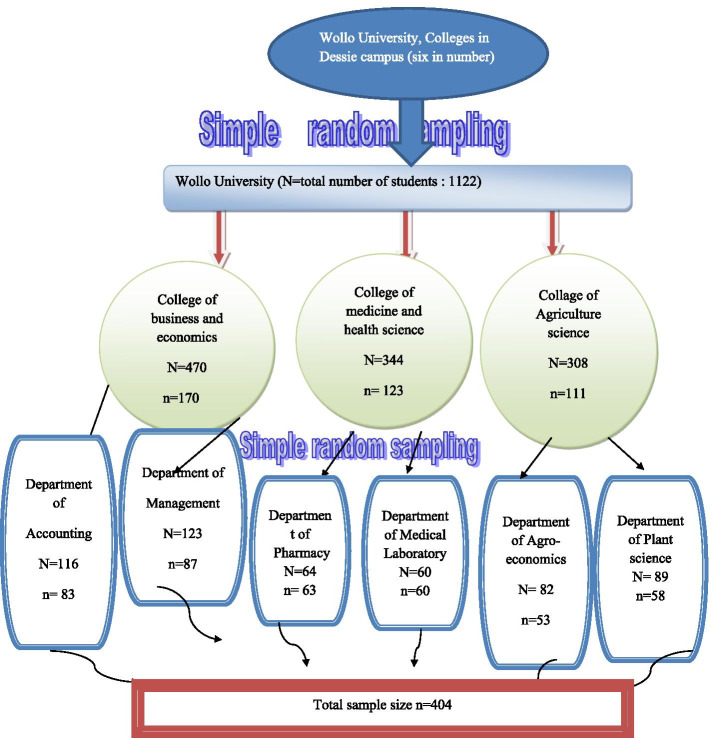
Schematic presentation of sampling procedure, Wollo University students, Ethiopia Nov 2020

There were a total of 1852 regular undergraduate students registered for the academic year of 2020, across different colleges. The sample size was determined using the single population proportion formula by taking the prevalence of migraine headache 13% [29], and 95% confidence interval and, 5% margin of error was used and finally sample size was 192. After adding a 5% none response rate the sample size was 202. Since we were used the multi-stage sampling technique we multiply our sample size by 2 and we got 404. A two-stage cluster sampling technique was employed to select the study participants. In the first stage by using a simple random sampling technique we choose three colleges out of the six colleges. In the second stage, again by applying a simple random sampling technique, we select 6 departments from each chosen college and on the final stage, we proportionally allocated the sample to select 404 participants (Fig. 1). We excluded students with visual impairment, those unable to fill the self-administered questionnaire due to illness, and extension students.

### Measurements

The dependent variable is migraine headache (Yes or No). The independent variable includes socio-demographic characteristics (age, sex, marital status, family history migraine headache, monthly income, living place before joining to campus), academic factors (Academic year, academic CGPA), other potential factors (stress, sunlight, noise, fatigue, heat, menstruation in women, too much work late sleep at night, exercise, smells and some foods) and mental illness-mood and emotional changes, suicidal ideation, and attempt.

### Operational definitions

#### Having migraine headache

From the Identity migraine test tool used, three questions having one or more symptoms on headache-related disability, nausea, and photophobia [38].

#### Migraine disability assessment questionnaire score (MIDAS)

Score 0–5 is grade 1 or little or no disability, score 6–10 is grade 2 or mild disability, score 11–20 is grade 3 or moderate disability, score 21 and above is the severe disability [39].

**Individual scores on the PSS** can range from 0 to 40 with higher scores indicating higher perceived stress. Scores ranging from 0 to 13 would be considered low stress, 14–26 would be considered moderate stress and 27–40 would be considered high perceived stress [40].

#### OSLO social support scale

A score ranging from 3 to 8 would be considered poor social support, 9–11 moderate social support, and 12–14 strong social support [41, 42].

### Data collection tools and instrument

The data was collected using a self-administered questionnaire which contains the socio-demographic variables and different tools were used (Identity migraine test) to measure migraine headache, and its disability (MIDAS), perceived stress (PSS), and level of social support (OSSS-3). Identity migraine testis the most widely used and validated of the migraine tools is the ID Migraine, with three questions on headache-related disability, nausea, and photophobia [38]. MIDAS is used to assign a disability grade indicating the overall intensity of illness during a 3 month recall period: little or no disability (grade 1, score 0–5), mild disability (grade 2 scores 6–10), moderate disability (grade 3, score 11–20), severe disability (grade 4 score 21 and above) [10, 39].

The Perceived Stress Scale (PSS) is a classic stress assessment instrument. The tool, while originally developed in 1983, remains a popular choice for helping us understand how different situations affect our feelings and our perceived stress. The questions in this scale ask about your feelings and thoughts during the last month. In each case, you will be asked to indicate how often you felt or thought a certain way. Which has ten questions, for each question has five alternatives [40]. The OSSS-3 consists of three items assessing the level of social support. It has been recommended for epidemiological and population-based surveys. Consequently, it can be considered a suited instrument for the current thesis [41, 42].

### Data collection techniques and quality control

Data were collected by two BSc psychiatry nursing professionals using a self-administered questionnaire having eight parts. The first part is socio-demographic characteristics, the second part is containing questions regarding academic achievement, the third part contains questions used to assess the presence of migraine headache, the fourth part contains questions used to assess migraine disability, the fifth part contains questions related to stress, the sixth part contain factors may intensify migraine headache, the seventh part contains questions related to social support, the eighth part contains questions related to mental health and substance.

We took many measures to assure the quality of data. Before proceeding to data collection the training was given for data collectors about the tools used and how to approach the respondents and to clearly explain the objectives and the purpose of the study. The questionnaire was pre-tested 1 week before the actual data collection time on 5% of the sample size on undergraduate students at Woldya University and appropriate modification was made. Finally, the questionnaire was written in simple and understandable language, using double data entry, and checked for completeness before data entry and incomplete data was discarded.

### Data processing and analysis

Data clean-up and cross-checking were done before analysis. Data were checked, coded, and entered in EPI data 3.1 version then it was exported to SPSS version 21 for analysis.

Both descriptive and analytical statistical procedures were utilized. Descriptive statistics like percentage mean and standard deviation were used for the presentation of demographic data and prevalence of migraine headache. Tables also were used for data presentation. Binary logistic regression was used to identify factors associated with migraine headaches. Variables with *P*-value of less than 0.25 were considered as a candidate for multivariate analysis. Multiple logistic regression models were fitted to control the possible effect of confounders and finally, the variables which had an independent association with migraine headache were identified based on AOR, with 95%CI and *p*-value less than 0.05.

## Results

### Socio-demographic characteristics of the respondent

We approached 404 participants, of these 371 respondents participated with a response rate of 91.8%. The mean age of the respondents was 23 years of age with the standard division of ±2.836. More than half of the respondents were male190 (51.2%), orthodox followers 255 (68.7%), Amhara by ethnicity 258 (69.5%), and single 337(90.8%), while 193 (52%) of them were grown in rural areas. Less than half of the students120(32.3%) visits a place of worship daily. The average monthly income was 770 ETB and the majority of the students 271(78.3%) had no family history of migraine headache (Table 1).

**Table 1:** Socio-demographic characteristics of regular under graduate WU students, Amhara region, north east Ethiopia, Dessie, November, 2020(*n* = 371)

Character		Frequency	Percent (%)
Age	20–25	334	90.0
	26–30	31	8.4
	> = 31	6	1.6
Sex	Male	190	51.2
	Female	181	48.8
Marital status	Single	337	90.8
	Others	34	9.2
Religion	Muslim	100	27.0
	Orthodox	255	68.7
	Others	16	4.3
Ethnicity	Amhara	258	69.5
	Oromo	55	14.8
	Tigre	41	11.1
	others (snnp,benshangul,harer,gam	17	4.6
Family history	Yes	99	26.7
	No	272	73.3
Living condition	Urban	178	48.0
	Rural	193	52.0
Frequency of worship	Daily	120	32.3
	2–3 times a week	106	28.6
	Weekly	94	25.3
	Less than weekly	28	7.5
	Never	23	6.2
Monthly pocket money	0–549	204	55.0
	550–1199	130	35.0
	1200–1999	17	4.6
	> = 2000	20	5.4

### Academic related characteristics of students

The majority of the students 233(62.8%) are in the third year, besides 74(19.9%) were in the fourth year and the rest 64(17.3%) are in the fifth year. Besides 78(21%), 64(17.3%), and 50(13.5%) are accounting, pharmacy, and plant science from the selected department respectively. Moreover more than half of 195(52.6%) students have a cumulative grade point of 3–3.5. (Table 2).

**Table 2:** Shows academic-related characteristics of regular undergraduate WU students, Dessie, November, 2020(n = 371)

Character		Frequency	Percentage (%)
Year of study	Third-year	233	62.8
	Fourth-year	74	19.9
	Fifth-year	64	17.3
Students department	Pharmacy	64	17.3
	Medical laboratory	57	15.4
	Management	74	19.9
	Accounting	78	21.0
	Agro economics	48	12.9
	Plant science	50	13.5
Students CGPA	3.6–4	46	12.4
	3–3.5	195	52.6
	2.5–2.9	98	26.4
	1–2.4	32	8.6

### Prevalence of migraine headache among students

**Fig. 2:**
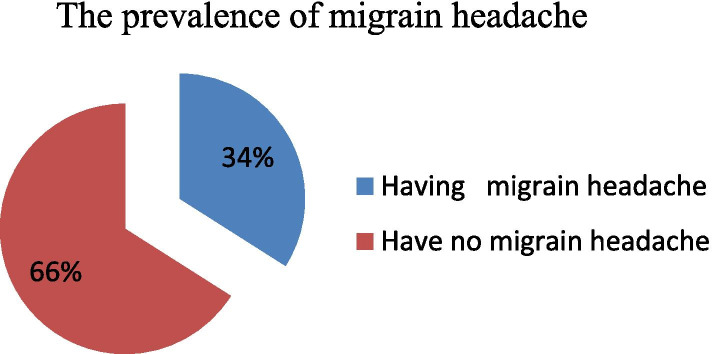
Prevalence of migraine headache among regular undergraduate WU students, Amhara region, northeast Ethiopia, Dessie, November, 2020(n = 371)

In this study, the lifetime prevalence of migraine headache among regular undergraduate WU students was 34% (95CI: 29.2, 38.5) (Fig. 2).

### Migraine disability assessment, perceived stress, and social support

**Fig. 3:**
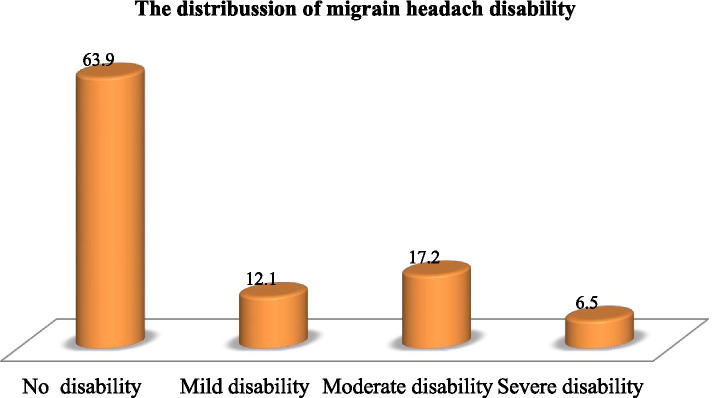
Assessment of migraine disability among regular undergraduate WU students, Amhara region, northeast Ethiopia, Dessie, November, 2020(*n* = 371)

**Fig. 4:**
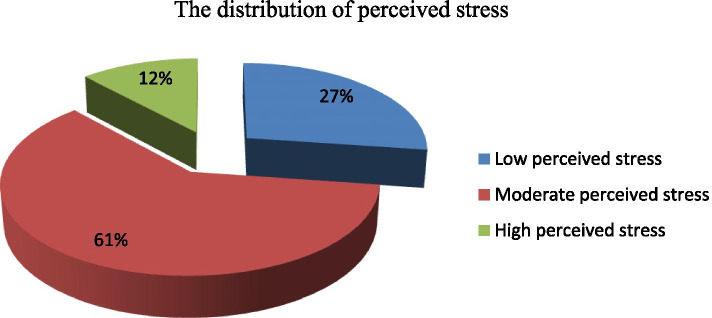
Assessment of perceived stress among regular undergraduate WU students, Amhara region, northeast Ethiopia, Dessie, November, 2020(n = 371)

**Fig. 5:**
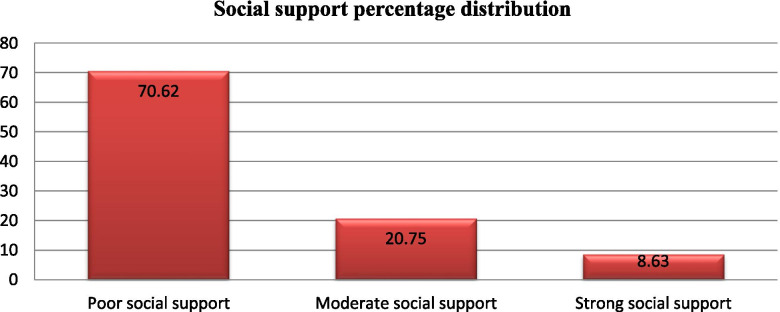
Assessment of social support among regular undergraduate WU students, Amhara region, northeast Ethiopia, Dessie, November, 2020 (n = 371)

Of the total participants, 237(63.9%) had no disability, 65(17.5%) had moderate disability, 45(12.1%) had a mild disability and the rest 24(6.5%) had severe disability. Similarly out of all participants 226(60.9%) of the students had moderate perceived stress, 101(27.2%) had low perceived stress, and 44(11.9%) had high perceived stress. Among study participants 262(70.62%) of the students had poor social support, 77(20.75%) had moderate social support and 32(8.63%) had strong social support (Figs. 3, 4 and 5).

### Factors triggering migraine headache

The most triggers reported for migraine headache were, noise 225(60.6%), sunlight 208(56.1%), fatigue (55.5%), too much work 205(55.3%), while late sleep at night 182(49.1%), smell 168(45.3%), and menstruation of women were 71(19.1%) (Table 3).

**Table 3:** Potential aggravating factors of migraine headache, among regular undergraduate WU students, Dessie,November, 2020 (n = 371)

Character		Frequency	Percentage (%)
Headache aggravated by exercise	Yes	133	35.8
	No	238	64.2
Headache aggravated by sunlight	Yes	208	56.1
	No	163	43.9
Headache aggravated by fatigue	Yes	206	55.5
	No	165	44.5
Headache aggravated by too much work	Yes	205	55.3
	No	166	44.7
Headache aggravated by noise	Yes	225	60.6
	No	146	39.4
Headache aggravated by menstruation (women)	Yes	71	19.1
	No	110	29.6
Headache aggravated by late sleep at night	Yes	182	49.1
	No	189	50.9
Headache aggravated by smells	Yes	168	45.3
	No	203	54.7
Headache aggravated by food	Yes	79	21.3
	No	292	78.7

### Assessments of substance and mental health

In our study 15(4%) of the students had a mental illness, 9(2.4%) had a history of suicidal attempts, 12(3.2%) had a suicidal thought in the last 3 months, 59(15.9%) had substance use (alcohol is the leading 27(7.3%)) and 17(4.6%) had a medical condition (Table 4).

**Table 4:** Assessments of substance and mental health among regular undergraduate WU students, Dessie, Amhara region, northeast Ethiopia, November 2020 (n = 371)

Character			Frequency	Percentage (%)
History of mental illness	Yes		15	4.0
	No		356	96.0
History of suicidal attempt	Yes		9	2.4
	No		362	97.6
Suicidal thoughts in the last 3 month	Yes		12	3.2
	No		359	96.8
Substance use history	Yes	Alcohol	27	7.3
		Khat	20	5.4
		Cigarettes	2	0.5
		Hashish	2	0.5
		Shisha	1	0.3
		Cannabis	2	0.5
		Others	5	1.3
	No		312	84.1
Diagnosed medical illness	Yes	HTN	1	0.3
		DM	0	0.0
		Epilepsy	5	1.3
		Others	11	3
	No		354	95.4

### Variables having a *p*-value less than 0.25 in bi-variable analysis

Variables statistically associated with migraine headache in bivariate analysis (*p*-value of < 0.25) was female students, marital status of other than single, Muslim in religion, having a family history of headache, never going to place of worship, monthly income of greater than or equal to 2000, having low perceived stress, and have suicidal thoughts. However, age, ethnicity, living residence before campus, study year, social support, and cumulative GPA of the students do not show any association with migraine (Table 5). Those variables with a *P*-value of < 0.25 in bivariate analysis were entered into multivariable logistic regression for further analysis to control the confounding factors. In multiple logistic regression analysis family history of migraine headache, history of suicidal thought in the past 3 months was significantly associated with migraine headache, while having low perceived stress positively affects the migraine headache.

**Table 5:** Bivariate analysis: Comparison of socio-demographic characteristics, social support, and perceived stress among regular undergraduate WU students, Dessie, November, 2020(n = 371)

Character		Migraine headache		COR(95% CI)	*P*-value
		Yes	No		
Age	20–25	115(31%)	219(59%)	Reference	
	26–30	10(2.7%)	21(5.7%)	.38(0.044, 3.299)	0.381
	> = 31	1(.3%)	5(1.3%)	.42(0.043, 4.087)	0.455
Sex	Male	71(19.1%)	119(32.1%)	Reference	
	Female	55(14.8%)	126(34%)	.73(0.475, 1.127)^a^	0.156
Marital status	Single	118(31.8%)	219(59%)	Reference	
	Others	8(2.2%)	26(7%)	.57(0.25, 1.3)^a^	0.18
Religion	Muslim	41(11.1%)	59(15.9%)	.68(.423, 1.09)^a^	0.116
	Orthodox	82(22.1%)	173(46.6%)	Reference	
	Others	3(.8%)	13(3.5%)	2.05(0.57, 7.4)	0.27
Ethnicity	Amhara	91(24.5%)	167(45%)	Reference	
	Oromo	17(4.6%)	38(10.2%)	1.28(0.473, 3.489)	0.623
	Tigre	11(3%)	30(8.1%)	1.565(0.509, 4.808)	0.43
	Others	7(1.9%)	10(2.7%)	1.9(0.582, 6.26)	0.286
Family history of headache	Yes	57(15.4%)	42(11.3%)	0.25(0.157, 0.406)^a^	0.00
	No	69(18.6%)	203(54.7%)	Reference	
Living residence	Urban	63(17%)	115(31%)	0.885(0.575, 1.36)	0.576
	Rural	63(17%)	130(35%)	Reference	
Frequency of worship	Daily	39(10.5%)	81(21.8%)	Reference	
	2-3timesaweek	36(9.7%)	70(18.9%)	0.733(0.268, 2.005)	0.545
	Weekly	31(8.4%)	63(17%)	0.686(0.249, 1.89)	0.467
	Less than weekly	14(3.8%)	14(3.8%)	0.717(0.257, 2.00)	0.525
	Never	6(1.6%)	17(4.6%)	0.353(0.107, 1.16)^a^	0.086
Monthly income	0–549	73(19.6%)	131(35.3%)	Reference	
	550–1199	40(10.8%)	90(24.3%)	0.598(0.209, 1.712)	0.338
	1200–1999	8(2.2%)	9(2.4%)	0.75(0.255, 2.2050	0.601
	> = 2000	5(1.4%)	15(4%)	0.375(0.093, 1.505)^a^	0.167
Study year	Third-year	76(20.5%)	157(42.3%)	Reference	
	Fourth-year	25(6.7%)	49(13.2%)	1.324(0.747, 2.346)	0.336
	Fifth year	25(6.7%)	39(10.5%)	1.256(0.627, 2.52)	0.52
Cumulative GPA	3.6–4	18(5%)	28(7.5%)	0.814(0.42, 1.578)	0.543
	3–3.5	67(18%)	128(34.5%)	Reference	
	2.5–2.9	32(8.6%)	66(18%)	1.08(0.645, 1.807)	0.771
	1–2.4	9(2.4%)	23(6%)	1.221(0.53, 2.815)	0.639
Mental illness	Yes	11(3%)	4(1%)	1.74(0.054, 0.557)^a^	0.003
	No	115(31%)	241(65%)	Reference	
Perceived stress	Low	18(4.7%)	83(22.4%)	3.166(1.782, 5.624)^a^	0.00
	Moderate	92(25%)	134(36.1%)	Reference	
	High	16(4.3%)	28(7.5%)	1.201(0.615, 2.346)	0.591
Social support	Poor	89(24%)	173(46.6%)	Reference	
	Moderate	24(6.5%)	53(14.3%)	1.33(0.628, 2.817)	0.456
	Strong	13(3.5%)	19(5.1%)	1.511(0.643, 3.551)	0.344
Have you ever attempt suicide	Yes	7(1.9%)	2(0.5%)	0.14(0.029, 0.684)^a^	0.015
	No	116(31.3%)	243(0.5%)	Reference	
In the last 3 month do you have suicidal thought	Yes	10(2.7%)	2(0.5%)	0.095(0.021, 0.443)^a^	0.03
	No	116(31.3%)	243(0.5%)	Reference	

^a^_Variable shows statistical significance associated with migraine headache on bivariate analysis_

### Factors independently associated with migraine headache

On the final model analysis, those who had a family history of headaches are more than 3.83 times (AOR = 3.83, CI: 2.313, 6.366) had a chance of having migraine headaches than those who had no family history of headache. Similarly, those who had suicidal thoughts in the past 3 months are more than 10.76 times (AOR = 10.76, CI: 2.117, 54.74) had a chance of having migraine headaches than, those who had no suicidal thoughts in the past 3 months. But on the other hand, those who had low perceived stress are 62.6% (AOR = 0.374, CI: 0.205, 0.683) less likely to have migraine headaches than those who had moderate and high perceived stress (Table 6).

**Table 6:** Multiple logistic regressions: factors independently associated with migraine headache among regular undergraduate WU students, Dessie, November, 2020(n = 371)

Character		COR	AOR	*P*-value
Sex	Male	Reference	Reference	
	Female	0.73(0.475, 1.127)	1.135(0.69, 1.855)	0.614
Marital status	Single	Reference	Reference	
	Others	0.57(0.25, 1.3)	2.714(0.981, 7.504)	0.054
Religion	Muslim	0.68(.423, 1.09)	0.667(0.386, 1.153)	0.147
	Orthodox	Reference	Reference	
	Others	2.05(0.57, 7.4)	1.66(0.42, 6.58)	0.469
Family History of headache	Yes	0.25(0.157, 0.406)	**3.837(2.313, 6.366)**^a^	0.00
	No	Reference	Reference	
Frequency of worship	Daily	Reference	Reference	
	2-3timesaweek	0.733(0.268, 2.005)	0.952(0.511, 1.772)	0.876
	Weekly	0.686(0.249, 1.89)	0.917(0.477, 1.766)	0.797
	Less than weekly	0.717(0.257, 2.00)	0.445(0.173, 1.14)	0.092
	Never	0.353(0.107, 1.16)	2.15(0.677, 6.852)	0.194
Monthly income	0–549	Reference	Reference	
	550–1199	0.598(0.209, 1.712)	1.333(0.784, 2.269)	0.289
	1200–1999	0.75(0.255, 2.2050	0.697(0.216, 2.25)	0.546
	> = 2000	0.375(0.093, 1.505)	1.138(0.338, 3.835)	0.835
Perceived stress	Low	3.166(1.782, 5.624)	**0.374(0.205, 0.683)**^a^	0.001
	Moderate	Reference	Reference	
	High	1.201(0.615, 2.346)	0.468(0.2, 1.095)	0.08
Mental illness	Yes	1.74(0.054, 0.557)	3.055(0.602, 15.5)	0.178
	No	Reference	Reference	
Have you ever attempt suicide	Yes	0.14(0.029, 0.684)	0.635(0.026, 15.29)	0.78
	No	Reference	Reference	
In the last 3 month do you have suicidal thought	Yes	0.095(0.021, 0.443)	**10.76(2.117, 54.74)**^a^	0.004
	No	Reference	Reference	

^a^Variables shown statistical significance associated with migraine headache on adjusted odds ratio

## Discussion

In our study, we tried to assess the prevalence, impact, and associated factors of migraine headache among regular undergraduate students in Wollo university. The prevalence of migraine headaches was high. Having a family history of headaches and had suicidal thoughts were the risk factors for migraine headaches, while had low perceived stress was the protective factor.

In our study, the prevalence of migraine headaches among regular graduate Wollo University students was found to be 34% at 95% CI (29.2, 38.5). This finding is higher as compared with the study conducted in Gondar university 13.06% [29, 43]. This may be due to different stressors like covid-19 epidemics during the data were collected. Likewise, in the study conducted inParakou Beninethe, lifetime prevalence was 14.3% (95% CI 12.3 to 16.4%) [22], in Zahedan among medical students was 7.14% [21, 42], in Greece, was 2.4% [18], and in Turkey was 12.4% [10]. This difference might be due to time variation, cultural and geographical differences, and environmental factors.

In our study the prevalence was slightly higher than the study conducted in Nigeria among nursing students was 26.8% [37], in Brazil, found a prevalence of 25% of migraines [10], and in King Abdul-Aziz University was 26.3% [37]. This might be due to sampling size differences, cultural and geographical differences, and environmental factors. However, our finding is in line with the study conducted in Kenya 33.8% [13]. In our study the MIDAS showed that 237(63.9%) had no disability (grade I), 65(17.5%) had a moderate disability (grade III), 45(12.1%) had a mild disability (grade II) and the rest 24(6.5%) had a severe disability (grade IV). This is consistent with an epidemiological study conducted in France, distribution of the MIDAS grade was found to be 74.7% grade I, 13.3% grade II, 7.7% grade III, and 4.3% grade IV [44].

In this study among the factors affecting the intensification of migraine headache, noise (60.6%), sunlight (56.1%), fatigue (55.5%), too much work (55.3%), late sleep at night (49.1%), smell (45.3%), exercise (35.8%), foods (21.3%) and in women menstruation (19.1%). For instance on the study conducted on Iran medical students the factors that had the highest effect on the intensification of migraine were fatigue (77.8%), stress (63%),sun light (55.6%),noise (48.1%), menstruation in women (55.6%), late sleep at night (40.7%),exercise(< 10%),smells like cigarette smoke and perfume odor (37%), and some foods like fatty (33.3%) [12].

Based on our study one of the factors that strongly associate with migraine is had a family history of headaches which increased the risk of migraine more than 3.83 times (AOR = 3.83, 95% CI: 2.313, 6.366) than those who had no family history of migraine headache. This is consistent with a study conducted at King Abdul-Aziz University [37]. But our finding is lower as compared with the study conducted in southeast China [10].

Moreover, in this study those who had suicidal thoughts in the past 3 months are more than 10.76 times (AOR = 10.76, 95% CI: 2.117, 54.74) had a chance of having migraine headache than those who had no suicidal thoughts in the past 3 months. Our finding was supported by the studies done in Taiwan [7]. However, our finding is somewhat high as compared with the study conducted in Rom [12]. This difference may be due to using a different type of method and geographical variation.

In this study having low perceived stress 61.4% (AOR = 0.386, 95% CI: 0.207, 0.72) were less likely to had migraines than those who had moderate and high perceived stress. This is consistent with the study conducted on Jazan medical students [20]. Besides in our study, we observed no relation to gender as in the study done in Iran-Zahedan, but there was a female predominance in most of the other studies in Riyadh, [36] in the USA, [12] in Nigeria, Brazil, and Turkey [9]. This difference may be due to using a different type of method, geographical variation, and cultural differences.

## Conclusion and recommendations

This study was aimed to assess the determinates of migraine headaches among regular undergraduate Wollo University students. Therefore the prevalence of migraine headaches was found to be very high. The predictors of migraine headaches were a family history of headaches, suicidal thoughts in the past 3 months, and had low perceived stress. So we recommend for different stakeholders, attention shall be given to those study participants who had a family history of migraine headache, suicidal thoughts, and high perceived stress.

## Strength and limitations

Conducting this study is important because as university students the curriculum requires constant concentration and hard work, students even a one-day absence of student can affect his/her school success, so we think that our research is important as it confirms the opportunity to evaluate migraine prevalence and its determinates, in a population where the disease could exert a relevant negative impact. The use of validated tools, doing multivariate logistic regression for analysis of data to control possible confounders could be our strength.

Besides this study had some limitations, which include using the cross-sectional method does not show cause and effect relationship. And also suicidal ideation and suicide attempts were evaluated using yes or no questionnaires rather than through psychiatric standardized evaluation tools. Furthermore, we did not classify migraine headaches, with aura and without aura. Therefore, we recommend further studies employing using psychiatric standardized evaluation tools to clarify suicide risk among patients with migraine headaches.

## Data Availability

The authors approved that the data supporting the conclusions of this article will be made available with the hand of Mengesha Birkie by (Email: mengeshasun@gmail.com) as per requested.

## Abbreviations

AOR: Adjusted Odd Ratio
CBE: Community Based Education
CGPA: Cumulative
ETTH: Episodic Tension-Type Headache
ETB: Ethiopian Birr
MIDAS: Migraine Disability Assessment score
HRQoL: Health-Related Quality of Life
ID Migraine: Identity migraine
SPSS: Statistical Package for Social Science
WU: Wollo University
WHO: World Health Organization
